# Spatiotemporal analysis of 3D human iPSC-derived neural networks using a 3D multi-electrode array

**DOI:** 10.3389/fncel.2023.1287089

**Published:** 2023-11-13

**Authors:** Doris Lam, Heather A. Enright, Jose Cadena, Vivek Kurien George, David A. Soscia, Angela C. Tooker, Michael Triplett, Sandra K. G. Peters, Piyush Karande, Alexander Ladd, Chandrakumar Bogguri, Elizabeth K. Wheeler, Nicholas O. Fischer

**Affiliations:** ^1^Physical and Life Sciences Directorate, Lawrence Livermore National Laboratory, Livermore, CA, United States; ^2^Engineering Directorate, Lawrence Livermore National Laboratory, Livermore, CA, United States

**Keywords:** 3D multi-electrode array, microelectrode array, neural networks, electrophysiology, 3D culture, hiPSC, collagen

## Abstract

While there is a growing appreciation of three-dimensional (3D) neural tissues (i.e., hydrogel-based, organoids, and spheroids), shown to improve cellular health and network activity to mirror brain-like activity *in vivo*, functional assessment using current electrophysiology techniques (e.g., planar multi-electrode arrays or patch clamp) has been technically challenging and limited to surface measurements at the bottom or top of the 3D tissue. As next-generation MEAs, specifically 3D MEAs, are being developed to increase the spatial precision across all three dimensions (X, Y, Z), development of improved computational analytical tools to discern region-specific changes within the Z dimension of the 3D tissue is needed. In the present study, we introduce a novel computational analytical pipeline to analyze 3D neural network activity recorded from a “bottom-up” 3D MEA integrated with a 3D hydrogel-based tissue containing human iPSC-derived neurons and primary astrocytes. Over a period of ~6.5 weeks, we describe the development and maturation of 3D neural activity (i.e., features of spiking and bursting activity) within cross sections of the 3D tissue, based on the vertical position of the electrode on the 3D MEA probe, in addition to network activity (identified using synchrony analysis) within and between cross sections. Then, using the sequential addition of postsynaptic receptor antagonists, bicuculline (BIC), 2-amino-5-phosphonovaleric acid (AP-5), and 6-cyano-5-nitroquinoxaline-2,3-dione (CNQX), we demonstrate that networks within and between cross sections of the 3D hydrogel-based tissue show a preference for GABA and/or glutamate synaptic transmission, suggesting differences in the network composition throughout the neural tissue. The ability to monitor the functional dynamics of the entire 3D reconstructed neural tissue is a critical bottleneck; here we demonstrate a computational pipeline that can be implemented in studies to better interpret network activity within an engineered 3D neural tissue and have a better understanding of the modeled organ tissue.

## 1. Introduction

Brain-on-a-chip (BOC) systems are engineered cell-culture models that allow non-invasive, real-time monitoring of electrochemical processes. Advances in cell-culture models now include 3D neuronal systems, including the addition of natural and/or molecules (ECM) to impart structural dimensionality, have improved the neuronal viability, neural network activity, drug responses, and resemblance to disease pathology compared to their 2D counterparts (Xu et al., 2009; Frega et al., 2014; Zhang et al., 2014; Bosi et al., 2015; Bourke et al., 2018; D’Aiuto et al., 2018). However, electrophysiological assessment of this 3D system has been technically challenging and is most often limited to planar multi-electrode arrays (MEAs), which detect networks solely at the bottom of the 3D construct (Frega et al., 2014; Bastiaens et al., 2018; Bourke et al., 2018), and patch-clamp electrophysiology, being accessible to only cells closest to the surface of the 3D biological system (O'Shaughnessy et al., 2003; Ma et al., 2004; Xu et al., 2009; Schaefer et al., 2019). A number of very promising technologies have been developed to address the obstacle of probing 3D neuronal models (Lam et al., 2021), including two recent prototypes of 3D Multi-electrode arrays (MEAs) that provide systematic and controlled interrogation throughout all planes of a 3D culture (Soscia et al., 2020; Shin et al., 2021). However, more functional studies are needed to evaluate the spatial precision of monitoring network activity from the 3D neural tissue before determining how closely the generated spatial patterns of neural activity recapitulate *in vivo* activity.

We have developed a novel prototype of a 3D MEA, in a bottom-up configuration (Lam et al., 2021), that interfaces directly with commercial MEA electrophysiology hardware (Multi Channel Systems MEA2100) without any additional modification (Soscia et al., 2020). Integrated with a 3D hydrogel-based neural tissue, we have demonstrated the unprecedented means to non-invasively monitor the extracellular field (i.e., single- and multi-unit spiking activity) generated from human iPSC-derived neurons co-cultured with primary human astrocytes (Soscia et al., 2020). In the present study, we use this same co-culture system to describe analytical methods used to assess the spatial precision of monitoring network activity from the 3D tissue-like structure during the development and maturation of neural and network activity, and when challenged by glutamatergic and GABAergic antagonists that affect synaptic transmission. Two main analytical approaches were used for the functional assessment of our human-based 3D system: (1) cross sectional analysis of the 3D MEA, taking the average level of activity from a pair of electrodes based on its vertical position on the probe of the 3D MEA; and (2) synchrony analysis of networks identified from active electrodes within or between cross sections. Using these two approaches, distinct growth and maturation patterns were observed for specific cross sections of the 3D neural tissue over the course of 45 days *in vitro* (DIV). When evaluating synaptic transmission, we detected region-specific networks and tracked its sensitivity to postsynaptic receptor antagonists, bicuculline (BIC, for GABA_A_ receptors), AP-5 (for NMDA receptors) and CNQX (for AMPA and kainate receptors). Collectively, we have developed a framework to experimentally and computationally analyze 3D neural network activity throughout a 3D hydrogel-based neural tissue, which can be adapted for future studies to evaluate disease or injury *in vitro* models and/or evaluate short- or long-term consequences of chemical and therapeutic agents on neural activity.

## 2. Materials and methods

### 2.1. 3D human neuron and astrocyte co-cultures

Human iPSC-derived neurons and primary astrocytes (Neucyte, San Carlos, CA) were encapsulated in ECM-collagen hydrogel and co-cultured on the 3D MEA devices, as previously reported (Lam et al., 2020; Soscia et al., 2020). All reagents were purchased from Thermo Fisher Scientific (Franklin, MA) unless otherwise stated. Briefly, concentrated rat tail collagen Type 1 (9.41 mg/ml, Corning, Bedford, MA) was diluted in seeding neuronal media (Neucyte) and the ECM mixture, Maxgel (200 μg/mL, Sigma-Millipore, St. Louis, MO), and neutralized to ~7.4 with 0.2N of NaOH. The ECM-collagen gel solution was chilled on ice to reduce collagen fibrillogenesis before the cell suspension was added. The cell suspension, in seeding media, contained human iPSC-derived neurons and primary astrocytes per vendor-recommended ratios: 70:30 glutamatergic to GABAergic neurons, and 75:25 for neurons to astrocytes, for a total of 5 × 10^6^ or 6.67 × 10^6^ cells/ml. The cell containing gel solution (75 μl at 3 mg/ml of collagen) flooded each array, and collagen fibrillogenesis occurred in a humidified incubator (37°C, 5% CO_2_) for 2 h. After 2 h, neuronal seeding media was added to wells and maintained in a humidified incubator (37°C, 5% CO_2_). After 24 h, cultures were maintained in short term media (Neucyte) for 1 week before switching to long term media (Neucyte) for the duration of the experiment. For culture maintenance, 50% of media was replaced every 2–3 days.

### 2.2. Chemicals

For electrophysiology recordings, stock solutions of the antagonists, bicuculine (BIC), DL-2-Amino-5-phosphonopentanoic acid (AP-5), and 6-Cyano-7-nitroquinoxaline-2,3-dione disodium (CNQX; all 5-7X concentrated), were prepared in dimethyl sulfoxide (DMSO) for BIC and CNQX or doubled distilled water for AP-5 (all from Tocris Bioscience, MO). Antagonists, either alone or in combination, were directly added to culture medium to provide final concentrations of 10 μM BIC, 50 μM AP-5 and 30 μM CNQX. For the vehicle-treated arrays, DMSO was added to match the final concentration of DMSO (0.037, 0.10 and 0.27%) in the chemical-treated group as a result of cumulative addition of BIC and CNQX.

### 2.3. Immunocytochemistry

The 3D cultures were fixed with 4% paraformaldehyde (1 h), washed in PBS (5 min, 3X), permeabilized with 0.2% Triton-x100 and 5% BSA (PBS-T,10 min), then blocked with 10% goat serum in PBS-T (4°C, overnight). All subsequent wash steps were conducted on an orbital shaker at 60 rpm. Cultures were washed in PBS-T (1 h, 3X) before labeling with primary antibodies for anti-class III beta-tubulin (Tuj1, chicken, 1:200, Neuromics, Edina, MN), and anti-Glial fibrillary acidic protein (GFAP, mouse, 1:200, Sigma-Millipore). Cultures were washed with PBS-T (2X for 1 h, 1X overnight) before incubating with secondary antibodies, Alexa Fluor 647-conjugated goat anti-chicken (1:200) and Alexa Fluor 488-conjugated goat anti-mouse (1,200). For nuclear staining, cultures were incubated in 4′-6-diamidino-2-phenylindole (DAPI; 1:3000). Samples were stored in PBS (dark, 4°C) before imaging using a LSM700 confocal microscope (Carl Zeiss Microscopy, Thornwood, NY).

### 2.4. Image acquisition and analysis of cell-containing 3D cultures

Optimal camera exposure and microscope settings were fixed for all replicates of ECM-Collagen gel samples on the 3D MEA. 2D images (1,024 × 1,025 pixels) in the XY plane at 2.5X and 10X magnifications (Zeiss Fluar 2.5x/0.12 M27, and EC PlanNeo 10x/0.30, respectively) were acquired at 10 μm intervals from the bottom of the gel (no fluorescence) to the top of the gel (disappearance of fluorescent signal). Sampling of 3D cultures included 2–3 regions of interest from each of the three replicates. To visualize cells and probes of the 3D MEA, Z-stack images of ECM-collagen gel samples were loaded into ZEN Blue (Carl Zeiss Microscopy), filtered using the binomial filtered method, and rendered into a 3D object in Orthogonal viewer with maximal intensity projection selected. As before (Thevenot et al., 2008; Lam et al., 2020), cell distribution was quantified by loading Z-stack images of DAPI-stained nuclei into Fiji (https://fiji.sc/#; National Institutes of Health, Bethesda, MD) and thresholded to remove any background fluorescence. The particle counter setting was adjusted for the particle size (in pixel units) with no adjustment in circularity, and all Z-stacks were scanned. The total particle number for each section was normalized to the dimensions of the field of view at 10X magnification to calculate the cell density per mm^2^.

### 2.5. 3D MEA, electrophysiology, and chemical exposure

The fabrication and design of the 3D MEA 3-well layout device were recently described (Soscia et al., 2020). Each device contains three arrays each within a well, and each array consisting of 10 flexible polymer (polyimide) probes that are vertically actuated to approximately 90° (8.8 to 12.7° probe-to-probe variability within an array; Soscia et al., 2020). Every polymer probe incorporates 8 thin-film electrodes, for a total of 80 electrodes per array and 240 electrodes per device. Prior to adding the cell suspension onto the 3D MEA, devices were plasma treated (PDC-001-HP, Harrick Plasma), sterilized with 70% ethanol (30 min), and rinsed with sterile distilled water. For electrophysiological recordings, the 3D MEA is placed within a 5% CO_2_-regulated chamber on the heated stage (37°C) of the 256-channel MEA2100 recording system (Multichannel Systems, Reutlingen, Germany). Recordings started after a 5-min equilibration time with an action potential spike defined by a lower limit threshold, set at 6.5×x the standard deviation of baseline noise, for each electrode. Each device was recorded for 30 min at a sampling frequency of 10 kHz and bandpass filtered between 4 and 4,000 Hz, as before by our group (Soscia et al., 2017, 2020; Lam et al., 2019; Enright et al., 2020) and others (Novellino et al., 2011; Pastore et al., 2018). Chemical exposure experiments were conducted at 45 DIV. Prior to addition of the chemicals, the culture media volumes were reduced to 100 μl. As described in previous 2D (Gramowski et al., 2006; Novellino et al., 2011; Scelfo et al., 2012; Mossink et al., 2021) and 3D (Shin et al., 2021) studies, devices were then equilibrated in the recording instrument for 20 min to minimize background signals from mechanical perturbations, to allow neural activity to stabilize, and/or to ensure diffusion of the chemicals before the 30 min recordings were conducted for baseline or chemical treatment. After baseline recording, arrays were randomly assigned for vehicle or chemical treatment. Chemicals were administered to the arrays in sequential order with BIC, AP-5, and CNQX. No washing was conducted between chemical treatments in order to evaluate the effects of fast GABA_A_R on NMDAR and AMDAR-dependent network activity, as before (Teppola et al., 2019). Due to the short half-life of BIC at pH 7.6 buffer (t_1/2_ = 45 min at 24°C; Olsen et al., 1975), 10 μM BIC was added to the AP-5 and CNQX solutions to ensure that ~10 μM BIC is present throughout the experiment. In vehicle group, the cumulative addition of BIC, increased the DMSO concentration from 0.037 to 0.10% and finally 0.27% with the addition of CNQX. Following each chemical addition, the devices were equilibrated in the instrument for 20 min, followed by a 30-min recording period.

### 2.6. 3D MEA data analysis

**Figure 1:**
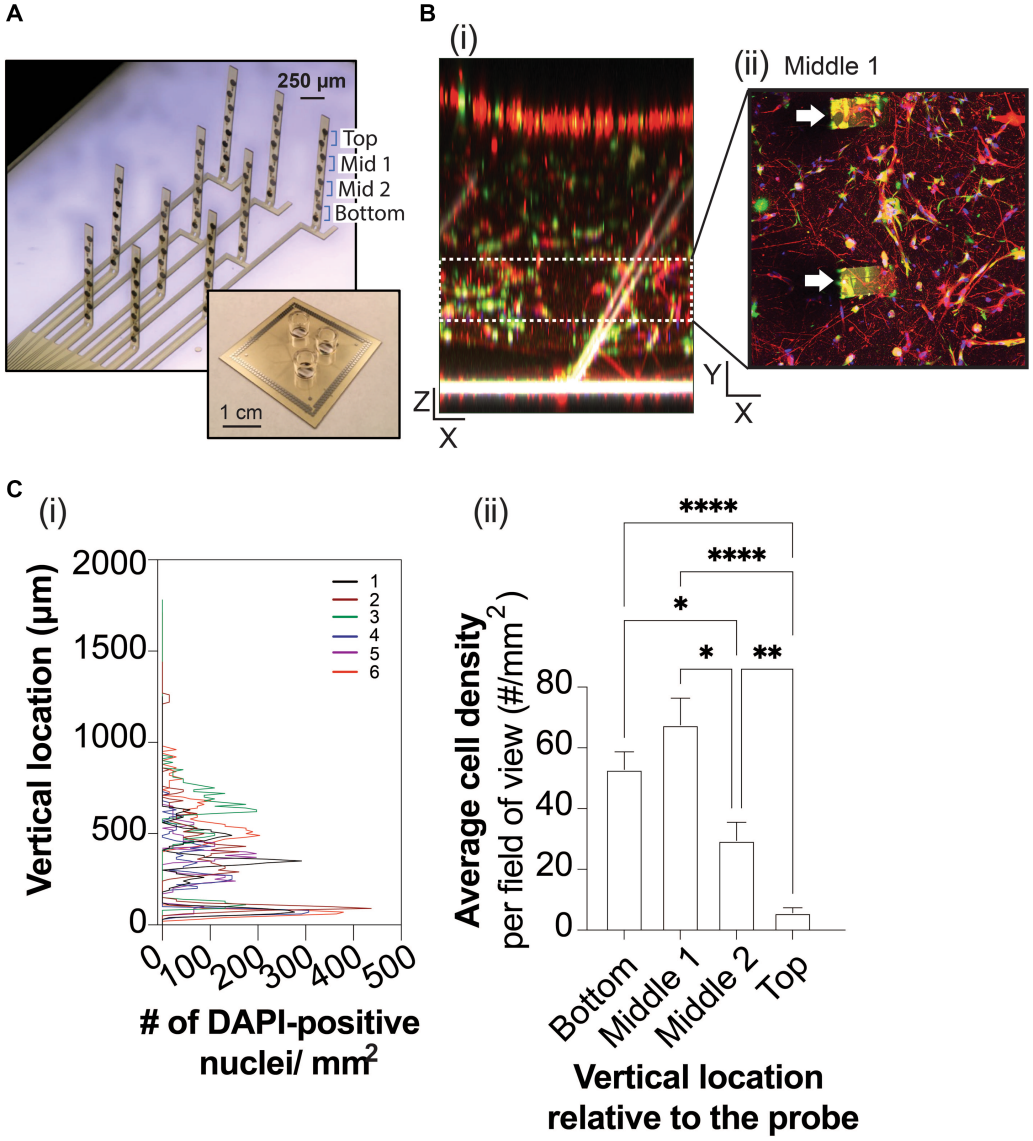
3D neuron and astrocyte co-culture grown on 3D MEA for 45 DIV. **(A)** A 3-well device (inset) allows simultaneous MEA recordings from three 3D cultures. Each well contains a 3D MEA that features 10 actuated probes to distribute 8 electrodes, which spans the height of 3D cultures. **(B)** Representative images of Tuj1-positive neurons (red), GFAP-positive astrocytes (green) and DAPI-positive nuclei relative to the flexible probes (white) of the 3D MEA, rendered into a 3D object in the XZ plane **(i)** and as a maximized projection image in the XY plane showing a cross section (e.g., middle 1) of the 3D co-culture, binned based on the height of a pair of electrodes embedded in the actuated probe **(ii)**, as labelled in **(A)**. Scale bars in the X, Y, and Z plane are 100 μm. White arrowheads indicate probes within the 3D culture in **(Bii)**. **(C)** Line graph **(i)** summarizes the total DAPI-positive nuclei counted (total nuclei/mm^2^) from the bottom to top of the ECM-Collagen gel for each technical replicate (colored line). The average DAPI-positive nuclei counted from Z-stack images (in the XY plane) were binned into cross sections based on the height of a pair of electrodes on a probe, **(ii)**. Data are displayed as mean ± SEM for *n* = 3 field of view from 3 wells and were analyzed using two-way ANOVA with Tukey’s *post hoc* test. Statistical significances were observed for cell distribution when comparing cross sections at a level of ^*^p < 0.05, ^**^p < 0.01, and ^****^p < 0.0001.

**Figure 2:**
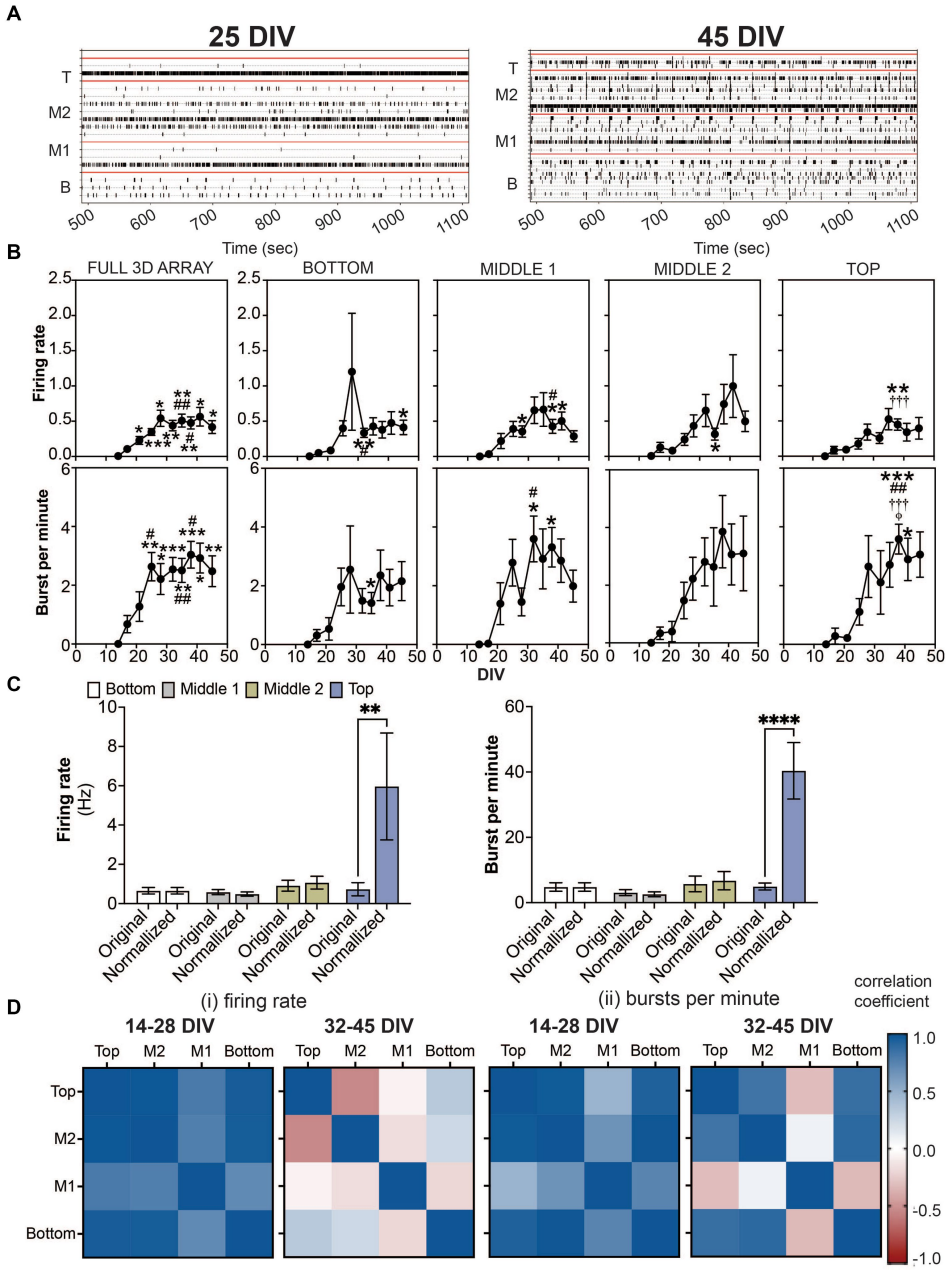
Detection of spikes and bursts within a 3D neuron-astrocyte co-culture monitored over 45 DIV. **(A)** Representative 10 min-raster plots of spiking and bursting activity within bottom, middle 1, middle 2 and top cross sections, delineated by the red line, of the flexible probes within the 3D MEA at 25 and 45 DIV. **(B)** Dot plots summarizes the firing rate (top row) and bursts per minute (bottom row) detected over the 30-min recordings. From left to right, graphs are shown with respect to the total activity across all electrodes within the (i.e., full 3D array) and sections of the 3D array based on electrode positions (i.e., bottom, middle 1, middle 2 and top). Data is presented as the mean ± SEM for *n* = 15 wells and were analyzed using mixed model of two-way ANOVA with Tukey’s *post hoc* test. Statistical significances (symbol) are observed for the feature of spike and burst activity when compared to 14 DIV (^*^), 17 DIV (^#^), 21 DIV (^†^), 25 DIV (^‡^), and 28 DIV (^φ^) at a level (number of symbols) of ^#^*p* < 0.05, ^##^*p* < 0.01, ^###^*p* < 0.001. **(C)** Bar graph compares the original data for the firing rate (left) and bursts per minute (right) for each cross section, and when the average data is normalized by the cell density within each cross section, determined from Figure 1C. Data is presented as the mean ± SEM for *n* = 15 wells and were analyzed using two-way ANOVA with Bonferroni’s *post hoc* test. Statistical significances is reported at a level of ^**^p < 0.01, ^****^p < 0.0001. **(D)** Correlation coefficient matrices display the strong positive (correlation score closer to 1.0) and negative relationships (score closer to −1.0) between layers (top, middle 2, middle 1 and bottom) of the 3D culture for the mean firing rate **(i)** and burst per minute **(ii)** grouped by the growth phase (development) of the active culture (14–28 DIV), and plateau (maturation) phase (32–45 DIV).

Time stamped data from each recording was exported as a hdf5 file and analyzed using an in-house custom R package to: (1) reconfigure the mapped electrodes in the MCS 256-channel format to the 80-channel format mapped for each of the three 3D MEA on the device, (2) remove silent electrodes (<10 spikes over recording period), (3) calculate spiking and bursting features of neuronal networks of each electrode for the 30-min recording (Soscia et al., 2017; Lam et al., 2019; Enright et al., 2020; Soscia et al., 2020); and (4) group the analyzed data by the known location of a pair of electrodes given its position on the probe. For each probe, “bottom” corresponds to electrodes 1 and 2, “middle 1” to electrodes 3 and 4, “middle 2” to electrodes 5 and 6, and “top” to electrodes 7 and 8 (Figure 1A). Burst detection parameters, were inspired by Chiappalone et al. (2006) and Charlesworth et al. (2015), and used previously (Soscia et al., 2017; Lam et al., 2019; Enright et al., 2020; Soscia et al., 2020). This include: maximum beginning interspike interval (ISI) of 0.1 s, maximum end ISI of 0.2 s, minimum interburst interval (IBI) of 0.5 s, minimum burst duration of 0.05 s, and minimum number of spikes per burst of 6. For electrodes within an array of a well that had no detectable spiking or bursting activity, a value of “0” was determined. For chemical exposure experiments, the mean and standard deviation (for a specific feature) prior to chemical exposure (e.g., baseline) was calculated. Then, the mean following each chemical exposure was calculated and normalized to baseline activity. The same calculations were determined for the vehicle-treated wells before and after DMSO exposure. To remove the effect observed with increasing DMSO concentration (Supplementary Figure 2), the values for the chemical-treated wells (normalized to baseline) are expressed as a fold change relative to the average value from the vehicle-treated wells (normalized to baseline) and used for further statistical analysis. Synchrony analysis was performed as previously described (Lam et al., 2019; Cadena et al., 2020), using the SPIKE distance (Kreuz et al., 2013) to measure pairwise synchrony between electrodes. The SPIKE-distance measures the dissimilarity between two spike trains as the average of the *instantaneous* dissimilarity between the two spike trains at different points of the recording. This measure has been previously used for quantifying synchrony in cultured hippocampal neurons (Zapata-Fonseca et al., 2016), understanding social cognition (Eisenman et al., 2015), and to estimate synaptic weights for training robot locomotion (Espinal et al., 2016). As in previous studies (Eisenman et al., 2015; Lam et al., 2019; Cadena et al., 2020; Enright et al., 2020), values were subtracted from 1 to obtain a similarity or synchrony measure, such that value of 1 represents completely synchronous firing and a value of 0 denotes asynchrony. Additionally, values were normalized by the SPIKE-distance obtained on randomly generated spike trains to compensate for the documented bias of SPIKE distance to assign higher synchrony values to denser spike trains. In Figure 2C we normalize the data based on the averaged distribution of cells over 6 wells across the z-axis. To attain the normalization constants, first, we partition the z-axis into 250 μm segments, where each segment corresponds to the electrode locations (bottom, middle 1, middle 2, Top) outlined in Figure 1A. Then, we calculated the density of the cell bodies per location and divided each of the location densities by the density of the bottom (as a reference location). Finally, to get the normalization constants we invert the constants to get fold-difference in cell density.

### 2.7. Statistical analysis

Quantified data are expressed as mean ± standard error of the mean (SEM) for the number of wells indicated, unless stated differently. For electrophysiology experiments and image analysis, the statistical significance was analyzed in GraphPad version 8 (GraphPad Software, San Diego, CA) using unpaired *t*-test, mixed model repeated measures for one-way or two-way ANOVA with Tukey’s post-hoc analysis.

## 3. Results

### 3.1. Physical characterization of 3D MEA and 3D human-based cultures

Encapsulated in an ECM-collagen scaffold, human iPSC-derived neurons and primary astrocytes were grown on custom 3D MEAs in a 3-well layout device (Soscia et al., 2020; Figure 1A) for up to 45 days *in vitro* (DIV). At 45 DIV, Tuj1-positive neurons and GFAP-positive astrocytes are observed throughout the XY plane, and throughout the XZ plane (Figures 1Bi,ii). To ensure proper spatial interpretation of the electrophysiological data obtained from the 3D MEA, we examined whether the vertical actuation angles of the 3D MEA probes within a well changed over time in culture. Confocal microscopy was used to measure the actuation angle of each 3D MEA probe based on the autofluorescence of polyimide polymer (Soscia et al., 2020). The probe angle could be readily measured from 3D rendering of z-stack images (Figure 1Bi) of wells before cell seeding and from 3D cultures at 45 DIV. 3D co-cultures minimally affected the actuation angle of the 10 probes on the 3D MEA, with probe actuation angles decreasing ~10° from the original positions before cell seeding (73.44 ± 11.87°, *n* = 9 wells) to the final positions once cultures were terminated at 45 DIV (63.89 ± 7.10°, *n* = 7 wells). Z stack images of 3D co-cultures have an average height of 1,354 ± 71 μm. Cell distribution was examined by detecting the nuclear stain DAPI (Lam et al., 2020; Figure 1Ci), binning Z-stack images into 4 sections (i.e., bottom, middle 1, middle 2, and top) based on the height of a pair of electrodes as determined by the angle of the probe at 45 DIV. Preferential distribution was observed within the bottom 50% of the 3D co-culture (Figure 1Cii), which agrees with denser projections from Tuj1-positive neurons and GFAP-positive astrocytes in the XZ plane and as shown in the middle section of the 3D co-culture (Figures 1Ci,ii), and reduced DAPI and cell staining in the top 50% of the 3D culture.

### 3.2. Functional characterization of the development and maturation of 3D neural networks

The onset of neural activity was variable across 15 3D MEA cultures. While only 6 out of 15 cultures exhibited sparse and sporadic spiking activity at 14 DIV, all wells exhibited spiking and bursting activity by 17 DIV which persisted up to 45 DIV. Representative raster plots shown in Figure 2A display active electrodes exhibiting neural firing throughout the 3D co-cultures at 25 DIV and 45 DIV. As before (Soscia et al., 2017; Lam et al., 2019; Cadena et al., 2020; Soscia et al., 2020; Lam et al., 2022), burst detection parameters, inspired by Chiappalone et al. (2006) and Charlesworth et al. (2015), (see Materials and Methods) were used for the quantification of features that describe spiking and bursting activity from the 3D MEA. For cross sectional analysis of the features of spiking and bursting activity within a 3D MEA culture (Figure 2B; Supplementary Figure 1), pairs of electrodes (out of 8 electrodes) were grouped and segregated based on their vertical position on the 3D MEA probe (e.g., bottom, middle 1, middle 2, and top as shown in Figure 1A) and delineated by the red line in the raster plot. As a comparison to the cross sectional analysis, we had also taken a global approach (Full 3D MEA), wherein the average activity across all active electrodes was calculated. Without positional information, the full 3D array analysis revealed that the mean firing rate and burst per minute displayed a significant increase in activity over the period of 45 DIV, having plateaued by 28 DIV for the mean firing rate and 25 DIV for the mean burst per minute. The stabilization of spiking and bursting activity was independent of the increasing number of active electrodes (shown in blue, Supplementary Figure 1) over the period of 45 DIV. For cross sectional analysis, spiking activity was observed as early as 14 DIV, from the bottom, middle 1 and middle 2 cross sections. By 17 DIV, all cross sections displayed spiking activity. The appearance of bursting activity was detected at 14 DIV in only the middle 2 cross section, and by 17 DIV all cross sections displayed bursting activity. The frequency of spiking and bursting activity plateaued, at a later time point compared to the global analysis approach, showing statistical significance relative to earlier time points (e.g., 14 or 17 DIV), at 32–35 DIV for the bottom and middle 1, and 38 DIV for middle 2 and top cross sections. Similarly, we observed a statistically significant number of bursts at later time points, 32 DIV for the middle 1, and 35 DIV for bottom and top cross sections. No difference was observed for the middle 2 cross section over the period of 45 DIV. Additional features of spiking and bursting can be found in the Supplementary Figure 1. Here, we demonstrate that cross-sectional analysis may provide more information about 3D neural networks that may be overlooked when using the global analysis approach.

Further analysis was conducted to evaluate the relationship of cell density and cross-sectional activity. Our approach was to generate a probability density function of cell density by cross-sectional region, using the quantified data in Figure 1C, and apply that to the rate of spiking and bursting activity. Data from the cross-sectional analysis at 45 DIV was normalized by a constant (see Materials and methods). By normalizing the feature of spiking and bursting activity to cell density, it is apparent that the top cross section, which had the least number of cells, had nearly 6 times more spiking and bursting activity (Figure 2C).

Based on the observed growth and plateau phase of spiking and bursting activity (from Figure 2B), we calculated the correlation coefficients (Figure 2D) to examine whether the amount of neural activity over the two time periods, 14–28 DIV (for the growth phase) and 32–45 DIV (for the plateau phase) was similar or different between layers. The top and bottom cross sections were similar in the rate of spiking and bursting during the development and maturation phase, having high cross correlation scores (values closer to 1). While rate of bursting activity in the middle 2 cross section was highly correlated to both the top and bottom activity during both phases, the rate of spiking activity in the maturation phase was negatively correlated to the top cross section. Interestingly for both the rate of spiking and bursting activity during the growth and plateau phase, the middle 1 cross section was most distinct (displayed by a negative or low cross correlation score) compared to the other cross sections.

**Figure 3:**
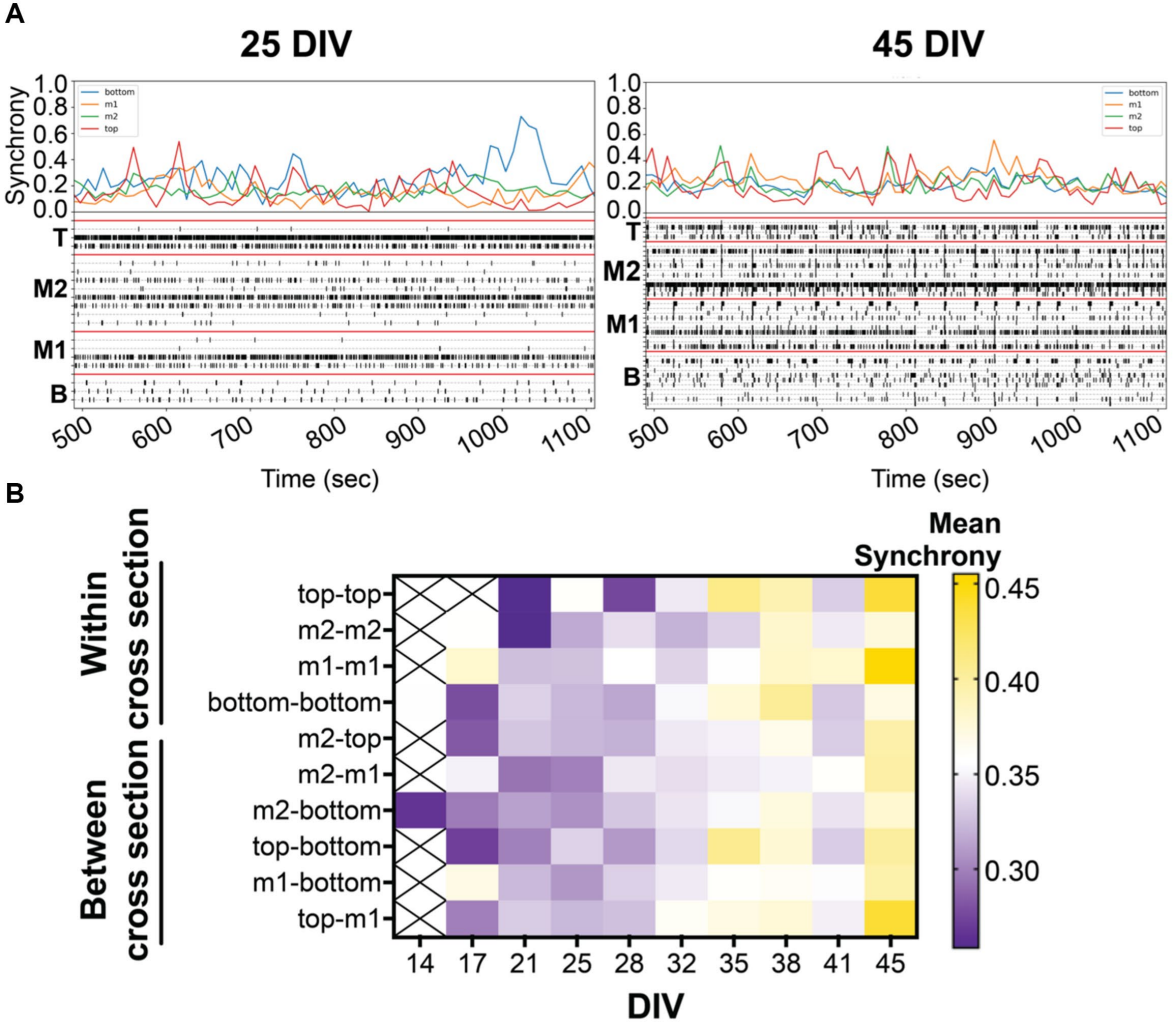
**(A)** Coordinated bursting activity is quantified by synchrony analysis (or 1-SPIKE distance), scoring a pair of active electrodes from 0 (asynchronous) to 1 (high degree of synchrony). Above the 10 min-raster plot (as shown in Figure 2) is the corresponding synchrony plot with the average synchrony scores over the 10-min recording period. Corresponding synchrony scores are reported for each cross section, bottom (blue), middle 1 (yellow), middle 2 (green), and top (red). Coordinated bursting activity is observed within cross sections, shown by the prominent peak, and between cross sections, shown by the alignment of peaks within the same time point. **(B)** The heat map illustrates the average synchrony value per array within (bottom, m1, m2, and top) and between cross sections over the period of 45 DIV.

Synchronized (or coordinated) bursting activity is a measure of mature networks in vitro (Kamioka et al., 1996; Voigt et al., 2001; Madhavan et al., 2007; Kuijlaars et al., 2016). In the present study, we examined the pairwise synchrony between active electrodes as described previously (Lam et al., 2019; Cadena et al., 2020). Scoring was determined from all possible electrode pairings within the 3D MEA based on specific criteria (see Methods) to establish whether the electrode pairs were synchronous or not. The comparisons generate a synchrony value (or 1-SPIKE distance) ranging from 0 (no synchrony) to 1 (high degree of synchrony) over the 30-min recording. An example is shown in Figure 3A where the average synchrony score is calculated from all possible electrode pairings within each cross section in the raster plot during 10-min snapshot. A higher synchrony score is observed when a large number of active electrodes within a cross section shows coordinated activity and is displayed by a prominent peak in the synchrony plot. Alignment of peaks across cross sections suggests coordinated activity between cross sections. Knowing the location of the synchronized networks (or the pair of electrodes) within the 3D MEA, we categorized the electrode pairs to look at network communication within and between cross sections. The degree of synchronization was shown to increase over the period of 45 DIV, as expected during the maturation process (Figure 3B). Interestingly, the highest synchrony scores were detected for networks within and between the top and middle 1 cross sections.

### 3.3. Evaluating synaptic transmission from 3D neural network activity following chemical exposure

**Figure 4:**
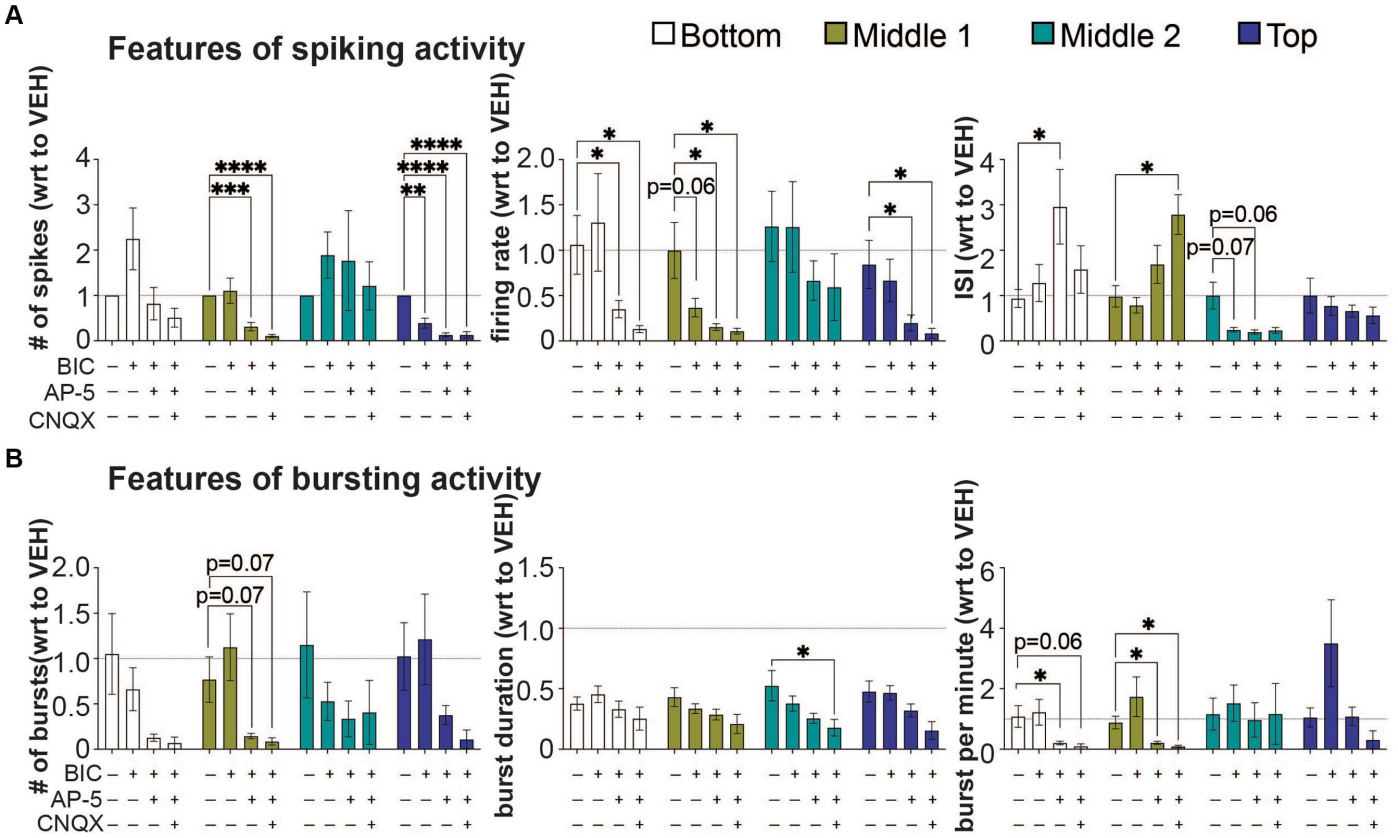
The effect of bicuculine, AP-5, and CNQX on features of spiking and bursting activity within a 3D neuron-astrocyte co-culture. 3D co-cultures at 45 DIV were untreated (baseline) and chemically challenged with the serial addition of BIC (10 μM), AP5 (50 μM), and CNQX (30 μM). **(A)** Features of spiking activity include number of spikes, overall firing rate, and interspike interval. **(B)** Features of bursting activity includes number of bursts, burst duration and bursts per minute. Because serial addition of the BIC and CNQX increased total DMSO concentration (from 0.037 to 0.27%) in the supernatant and have some effect on some features of spiking and bursting activity (see Supplementary Figure 2), data is expressed as a fold change (mean ± SEM for *n* = 9 wells) with respect to (wrt) the vehicle group (dotted line at 1). Data was analyzed using two-way ANOVA with Dunnett’s *post hoc* test. Statistical significances are observed when compared to baseline (*). The level of significance is denoted as ^#^*p* < 0.05, ^##^*p* < 0.01, ^###^*p* < 0.001, and ^####^*p* < 0.0001.

We evaluated the response of neural networks to postsynaptic receptor antagonists, using the features analyses of spiking and bursting activity (Figure 4). Glutamate and GABA should be the primary means for synaptic transmissions within the 3D neural tissue. The sequential addition of postsynaptic receptor antagonists for GABA_A_ receptors (BIC), NMDA receptors (AP-5), and AMPA and kainite receptors (CNQX) was applied to the 3D neural tissue to inhibit first GABAergic neurotransmission followed by glutamatergic neurotransmission (Figure 4A). Inhibition of GABAergic neurons, induced by GABA_A_ receptors antagonist, BIC, was shown to increase the synchronization of neural activity in 3D hydrogel-based neural tissue (Ming et al., 2020; Dingle et al., 2021), resulting from the disinhibition of excitatory neurons; thereby increasing overall activity within the 3D culture. Following the application of BIC, a decrease in spiking activity was detected in only the top cross section (number of spikes, *p* < 0.01), suggesting a greater presence of BIC-sensitive GABAergic neurons within this cross section of the 3D neural tissue. GABAergic mediated disinhibition of excitatory neurons was apparent by the increase in spiking activity (e.g., # of spikes and firing rate) within the bottom and middle 2 cross sections of the 3D neural tissue (Figure 4A) but was not statistically different to the vehicle-treated group (dotted line). No significant differences were observed within cross sections for features of bursting activity following BIC treatment (Figure 4B).

The subsequent application of the NMDA-receptor antagonist, AP-5, and CNQX, a competitive antagonist that acts on non-NMDA receptors AMPA and kainate-type of glutamate receptors, were applied in the presence of BIC, to evaluate which of these ionotropic glutamatergic receptors contributed to the remaining neurotransmission within the 3D neural tissue. The application of AP-5 (with BIC) revealed that there were cross sections in which NMDA receptors primarily mediate glutamatergic neurotransmission, blocking 70–90% of BIC-induce spiking activity in the middle 1 and top cross sections (based on number of spikes and firing rate). In these cross sections, AMPA receptors had a limited contribution to glutamate neurotransmission with either reducing the number of spikes and firing rate further or had no effect. Interestingly, the bottom and middle 2 cross sections revealed some degree of insensitivity to AP-5, having no change in the number of spikes and/or firing rate relative to vehicle-treated cultures, but either showed an increase (bottom cross section) or decrease (middle 2 cross section) in the interspike interval. The role of glutamatergic neurotransmission in regulating bursting activity within cross sections appeared to be highly variable showing a trend towards a decrease across all cross sections during AP-5 and CNQX applications (Figure 4B), and specific cross sections showing a significant decrease in the bursts per minute (i.e., bottom and middle 1), and burst duration (middle 2). In some cross sections, significant amount of neural activity remained following co-application of BIC, AP-5 and CNQX, suggesting that neurotransmitters other than GABA and glutamate were involved in regulating synaptic transmission within this 3D culture system.

### 3.4. Tracking the change in 3D network activity across chemical treatments

**Figure 5:**
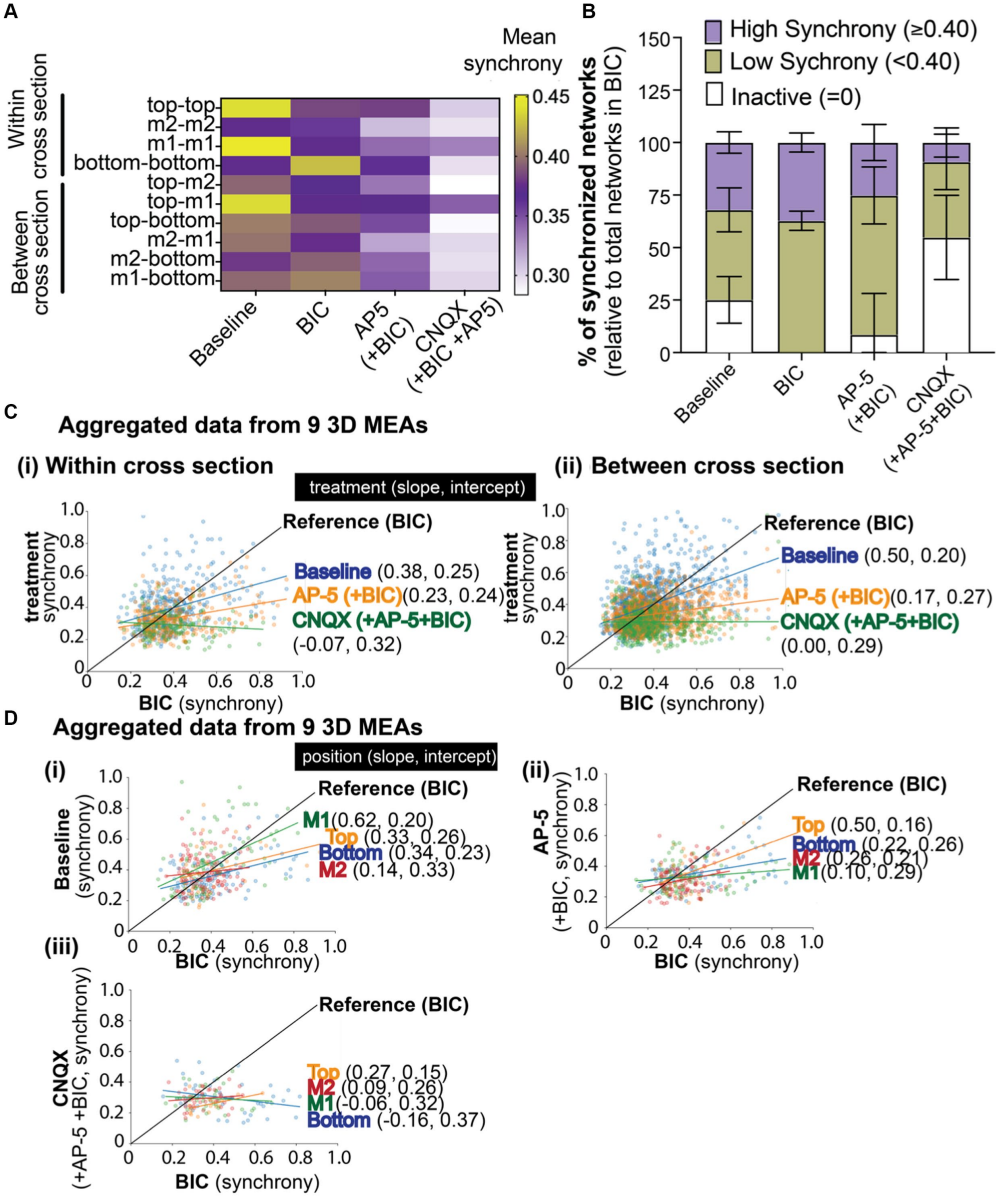
The effect of BIC, AP-5, and CNQX on synchronized neural network activity within a 3D neuron-astrocyte co-culture. **(A)** The heat map illustrates the average synchrony value per array within (bottom, middle 1, middle 2, and top) and between cross sections before and after the sequential addition of BIC, AP-5, and CNQX. **(B)** Overlay bar graph compares the average number of synchronized networks (or edges) detected within the 3D culture, independent of the electrode’s position, across treatment conditions (e.g., baseline, BIC, AP-5 + BIC, and CNQX+AP-5 + BIC). Data was normalized to the total edges identified during BIC treatment (see Results for rationale). Edge activity has been categorized by the degree of synchrony: “high synchrony” has a 1-SPIKE distance ≥ 0.40 (purple), “low synchrony” has a 1-SPIKE distance <0.40 (yellow), or inactive electrodes (white). Data is shown as mean ± SEM. **(C)** Scatter plot illustrates the shift in synchrony value for an active edge (dot) detected in BIC treatment (Reference, black line), its value before (e.g., baseline,), and after (e.g., AP-5 and CNQX) BIC treatment. The line of best fit was determined based on all available edges, categorized by the location within **(i)** or between **(ii)** cross sections, and the slope and intercept reported for each antagonist in brackets. **(D)** Scatter plot illustrates the shift in synchrony value for an active edge detected in BIC treatment (Reference, black line) based on its cross sectional position (e.g., bottom, middle 1, middle 2, and top graph) for baseline **(i)**, AP-5 **(ii)**, and CNQX **(iii)**. The slope and intercept are reported based on the line of best fit for each cross section. Electrode data has been aggregated for 9 3D MEAs.

The sequential addition of BIC, AP-5, and CNQX together with the electrode identification on the 3D MEA can allow researchers to track the sensitivity of the same network across multiple chemical exposures (Figure 5), as shown in this study. Cross sections with the highest level of synchrony were within and between the top and middle 1 cross sections, and networks from these regions were sensitive to BIC, decreasing its synchrony scores following the application of the antagonists (Figure 5A). A noticeable increase in synchrony scores were observed following BIC for any networks within the bottom cross section or those that projected from this region. The addition AP-5 to BIC-treated cultures for most cross sections showed a decrease in synchrony values for networks within and between regions of 3D culture, with the exception of networks within the top cross section and between top and middle 1 sections which were insensitive to the antagonist. CNQX appeared to have a greater effect on synchronized bursting activity, further reducing synchrony scores. Interestingly, networks within middle 1 and between top and middle 1 cross sections appeared to be less sensitive to CNQX, suggesting these regions in particular were regulated by other neurotransmitters.

Next, we evaluated whether we could track the changes in synchronized networks or those that were or became inactive during the chemical exposures. At a gross level (Figure 5B), indiscriminate of the location of the network within the 3D culture, networks (or edges) detected from a pair of electrodes from each array were grouped based on its synchrony (1-SPIKE distance) score: a score ≥0.40 was considered “high,” <0.40 was considered “low,” and inactive. The total number of edges (or networks) detected for each treatment condition and grouped score (e.g., high, low, or inactive) was normalized to the total edges detected from BIC-treated cultures, with the assumption that all active excitatory networks are available following BIC-induced GABAergic disinhibition (mediated by blocking GABA_A_ receptors. We found that network activity at baseline exhibited a relatively even distribution of higher levels of synchronized networks (1-SPIKE distance values ≥0.40, 32.0 ± 5.1% of total edges), lower levels of synchronized networks (<0.40, 42.8 ± 10.5% of total edges), and networks that were inactive (25.1 ± 11.1%) but became active with BIC treatment). Application of BIC increased the proportion of networks with low levels of synchronization (62.8 ± 4.6%). The addition of AP-5 inactivated a small proportion of networks within the high and low categorization of synchrony scores. Of the networks that remained, the following addition of CNQX inactivated nearly half of the networks within the high and low categorization of synchrony scores, suggesting that AMPA and kainate receptors, rather than NMDA receptors, was important for regulating the coordinating bursting activity.

To increase the resolution of the data analysis, we examined the shift in synchrony score at a network level based on its position within a cross section (Figure 5Ci) or between cross sections (Figure 5Cii). The scatter plot illustrates the shift in synchrony value for a network (dot) detected in BIC treatment (Reference, black line), its value before (e.g., baseline,), and after (e.g., AP-5 and CNQX) BIC treatment. The line of best fit was determined based on all available networks, categorized by the location within (top graph) or between (bottom graph) cross sections, and the slope and intercept reported for each antagonist in brackets. Most networks within and between cross sections clustered around a synchrony score of 0.4, suggesting a “homeostatic” level of synchronization within the 3D system. For baseline activity, networks (or blue dots) above the BIC reference line are likely those that include a GABAergic neuron; showing a high synchrony value at baseline that then decreases with BIC treatment. Networks below the reference line are likely excitatory networks; showing a lower synchrony value at baseline that increased with BIC treatment as a result of GABA disinhibition. Data points that lie above the BIC reference line after the addition of AP-5 are networks insensitive to the agonist. While networks sensitive to the NMDA antagonist showed a decrease in synchrony scores and are below the BIC reference line. The remaining networks, following treatment with AP-5 and BIC, sensitive to CNQX showed a further decrease in its score, and in most cases below “homeostatic” levels. Slopes of the treatment conditions were found statistically significantly different than BIC (see Supplementary Table 1).

We applied this method of tracking the shifts in synchrony scores to the position of the network within a specific cross section (Figure 5D) before (e.g., baseline, i) and after exposure to postsynaptic receptor antagonists [e.g., AP-5 (ii) and CNQX (iii)]. In Figure 5D, networks within the middle 1 cross section, at baseline (Figure 5Di), displayed a wide range of synchrony scores that were most sensitive to BIC application, showing more networks with scores that had decreased (left of the BIC reference line) compared to networks that increased its synchrony score (right of the reference line). Thus, resulting in a higher slope value compared to other cross sections. Top, bottom, and middle cross sections had shown similar slope and intercept values suggesting that the effect of BIC on the synchronized networks within these cross sections of the 3D hydrogel-based neural tissue were similar. The addition of AP-5 to BIC treatment (Figure 5Dii) revealed that most networks in middle 1 were sensitive to the antagonist, followed by middle 2, bottom, and top, being the least sensitive. The subsequent addition of CNQX (Figure 5Diii) revealed that networks within all cross sections had shifted its synchrony scores, as shown by the change in the slope, being close to 0 or a negative value.

## 4. Discussion

In the present study, we demonstrate that analytical methods are needed to improve the interpretation of the 3D network topology. These methods include (1) cross sectional analysis of the 3D neural tissue (based on the vertical electrode position of the 3D MEA), used to identify the differences in the development and maturation of neural activity; and (2) synchrony analysis, used to describe network activity within and between cross sections, and track its sensitivity in the presence of GABA and glutamate postsynaptic receptor antagonists.

Our 3D neural tissue was established (Lam et al., 2020; Soscia et al., 2020) using the “entrapment” method to encapsulate cells (i.e., human-iPSC derived neurons and primary astrocytes) in a pre-gel solution, allowing cells to be localized throughout the fibrous networks of collagen during fibrillogenesis (O'Connor et al., 2001; O'Shaughnessy et al., 2003; Ma et al., 2004; Xu et al., 2009). While the collagen scaffold does not mirror composition of extracellular matrix molecules within the brain, it is a natural material that is widely used for 3D neuronal cell culture (O'Connor et al., 2001; O'Shaughnessy et al., 2003; Ma et al., 2004; Xu et al., 2009; Sood et al., 2016; Kim et al., 2017; Bourke et al., 2018; Langhans, 2018; Tang-Schomer et al., 2018; Sorushanova et al., 2019; Shin et al., 2021). In previous work, we optimized the seeding parameters to improve upon cell viability and cell distribution of primary rat neurons within the scaffold, which was dependent on atmospheric CO_2_ and the initial cell concentration encapsulated (Lam et al., 2020). In the present study, we adapted the seeding protocol to encapsulate human iPSC-derived neurons and primary astrocytes within the collagen scaffold but found that cell distribution was heterogenous; predominantly distributed within the bottom half of the 3D biological system at 45 DIV (Figure 1). While it is unclear from this study whether species differences attributed to the difference in cell distribution, we have previously shown that there is a threshold to which cell concentration (e.g., 1 × 10^7^ primary rat brain cells/ml) has a mechanical impact on the collagen fibers after fibrillogenesis, also observed in fibroblast studies that have examined cell-collagen networks (Evans and Barocas, 2009; Fernandez and Bausch, 2009; Iordan et al., 2010). Despite the predominant distribution of cells at the bottom of the gel, we were able to detect neural activity across all 8 vertical electrode positions on the flexible probes (Figure 2).

The cross-sectional analysis of the 3D electrophysiology data over the period of 45 DIV was conducted to address whether the difference in cell density (i.e., bottom-heavy) affected the properties of networks formed and matured within the 3D hydrogel-based neural tissue. In our 3D tissue, the onset of spiking and bursting activity and the functional maturation of neural activity occurred earlier for the bottom two cross sections (e.g., bottom and middle 1), cross sections shown to have the highest cell density, compared to the top and middle 2 cross sections (Figure 2). Using synchrony analysis, networks with a low degree of synchronization were formed within the bottom, middle 1 and middle 2 or projected from these regions, suggesting that short- and long-range connections were formed earlier on (i.e., 17 DIV, Figure 3). It was previously reported that short distance networks between adjacent neurons are formed first in a 3D hydrogel-based neural tissue, and that these networks become relatively large as they mature, decreasing the number of networks while connections per network increased (Shin et al., 2021). It is possible that the difference in the observation depends on the type of analysis being conducted. However, using the cross-sectional and synchrony analyses in our 3D neural tissue, we report that the functional differences in the features of neural activity and the synchronization of networks observed between cross sections suggests that the density of cells distributed within the 3D tissue may also play a role in promoting the formation of short- and long- range neural networks, in addition to the maturation of these networks. In previous 3D (Ming et al., 2020; Vernekar and LaPlaca, 2020; Shin et al., 2021) and 2D culture studies using electrophysiology or optical recordings (Kamioka et al., 1996; Opitz et al., 2002; Chiappalone et al., 2006; Ito et al., 2010) the seeding density is recognized to affect neural network formation. Interestingly, in our study, the cross section with the least number of cells (i.e., top) was shown to have the greatest amount (~6-fold difference) spiking and bursting activity, and higher synchrony scores compared to the other cross sections. However, it is unclear whether this difference is attributed to the microenvironment of each cross section (e.g., diffusion or availability of nutrients, remodeling of the ECM, cell-to-cell contact) or the interaction of short- and long-range networks. Early work by Frega et al. (2014) reported regional differences in neural network activity in a 3D hydrogel-based neural tissue, comparing networks at the bottom of the tissue (using a planar MEA) and the top (using patch-clamp electrophysiology; Frega et al., 2014). Despite controlling for a uniform seeding density for each monolayer in the tissue, the authors attributed the regional differences to synaptic reverberation and amplification formed from a larger sub-network of neurons within the bottom layer (but not top layer). However, it is not clear if this is the case in our 3D neural tissue that has a “bottom heavy” distribution of cells.

Similar to Li and van den Pol (2009), Hanus et al. (2016), and Teppola et al. (2019), network activity dynamics were evaluate by sequential and acute application of GABA_A_, NMDAR, and AMPAR antagonists, applied to the whole cell culture (Figures 4, 5). Here, we examined whether the expected seeding ratio of glutamatergic to GABAergic neurons (75:25) is the same across layers by examining the sensitivity of the functional networks to postsynaptic receptor antagonists. If the seeding ratio was consistent across cross sections, then network activity dynamics would exhibit a similar change in response to the postsynaptic receptor antagonist. The sequential addition of GABA_A_, NMDAR, and AMPAR antagonists allowed us to show, for the first time, the tracking of 3D neural and network activity from the same electrode(s) after each addition of the antagonist (Figure 5) known to block GABA and glutamatergic neurotransmission. A similar approach was recently shown for 2D neural networks (Tomagra et al., 2023). Blocking GABA_A_ receptors and subsequently inhibiting GABAergic neuron activity with BIC is known to increase the excitability within the 3D hydrogel-based neural tissue (Ming et al., 2020; Dingle et al., 2021), a consequence of GABAergic-mediated disinhibition of excitatory neurons. In our 3D neural tissue, networks within the middle 1 and top cross sections suggests a functional trend that supports the presence of GABAergic neurons within these sections. The expected decrease in spiking activity (Figure 4) and synchrony scores for networks that projected from these regions (Figures 5A,D) were detected following the application of BIC. Interestingly, the functional response to BIC-sensitive networks within these cross sections were different in response to AP-5, the NMDA antagonists (Figures 5A,D). Specifically, a greater role of glutamatergic synaptic transmission contributed to network activity within the middle 1 cross section (sensitive to AP-5) and less so for networks within the top cross section (insensitive to AP-5). Further, networks that projected within and from the bottom cross section suggests a functional trend that supports GABAergic-mediated disinhibition of excitatory neurons. The expected increase in synchrony score was observed following the application of BIC, and a decrease in its score following the sequential addition to AP-5 (Figures 5A,D), confirming the role of glutamatergic synaptic transmission in these networks. The addition of CNQX, an AMPA and kainate receptor antagonist, inactivated nearly 50% of synchronized networks detected in the BIC application (Figure 5B), and affected all cross sections to the same degree (Figure 5D). This expected response was similar to previous studies that have applied NMDA receptor and AMPA and kainite receptor antagonists to 3D neural tissue (Sakaguchi et al., 2019; Trujillo et al., 2019; Ming et al., 2020; Shin et al., 2021). Collectively, the functional response of neural networks to the chemical exposures suggests an unbalance in the neuronal subtypes across cross-sections (using cross-sectional analysis for features of spiking and bursting activity) that forms complex networks within and between cross sections (using synchrony analysis) that cannot simply be described by the original seeding density ratio or cell density. Certainly, future studies will need to elucidate whether the timing of collagen fibrillogenesis or physical properties of collagen may affect the distribution of neuronal subtypes (using immunocytochemistry) and additional computational methods to assess the functional properties of 3D neural networks observed in this study (e.g., functional-effective connectivity; Pastore et al., 2018).

We demonstrate, for the first time, that the combination of the high spatial resolution offered by the 3D MEA and the sensitivity of the computational tools can track and quantify the change in synchronicity within subpopulations of neural networks (within and between cross sections), networks that are sensitive/ insensitive to chemicals, and those that are or become inactive as a result of chemical exposures. However, there are limitations to the experimental and computational methods proposed that should be considered in future studies, which includes: (1) the dependence on how “high” vs. “low” levels of synchronized networks are defined (i.e., threshold based on 1-SPIKE distance values); (2) utilizing chemicals with clearly delineated inhibitory mechanisms; and (3) if mechanical disturbance by applying the antagonists or the diffusion rate of the antagonists across the cellular layers is a confounding variable to consider.

With the rapid rise in the development of 3D reconstructed neural tissues, there is the demand for the electrophysiological assessment of these biological systems and the computational tools to interpret the temporal dynamics and spatial configuration of the networks, as well as the directionality of network communication, thereby providing feedback to the researcher on whether the construct of the 3D tissue needs to be optimized. Here, we demonstrate the ability to monitor the spatial dynamics of 3D human neural network activity using our 3D MEA, which facilitates network analysis based on the 3D position of the electrodes, thus enabling the measurement and analysis of temporal changes in activity throughout the tissue and providing a greater depth of understanding of the functional 3D network properties. The spatiotemporal resolution and sensitivity of these measurements highlights the utility of 3D MEAs to advance basic scientific and translational research using 3D engineered neuronal cultures.

## Data availability statement

The original contributions presented in the study are included in the article/Supplementary material, further inquiries can be directed to the corresponding authors.

## Ethics statement

Ethical approval was not required for the studies on humans in accordance with the local legislation and institutional requirements because only commercially available established cell lines were used.

## Author contributions

DL: Conceptualization, Data curation, Formal analysis, Funding acquisition, Investigation, Methodology, Resources, Writing – original draft, Writing – review & editing, Validation. HE: Conceptualization, Methodology, Supervision, Writing – review & editing, Resources. JC: Methodology, Writing – review & editing, Formal analysis, Validation. VG: Methodology, Writing – review & editing, Formal analysis, Validation. DS: Methodology, Resources, Writing – review & editing. AT: Methodology, Writing – review & editing, Resources. MT: Methodology, Writing – review & editing, Resources. SP: Methodology, Validation, Writing – review & editing. PK: Formal analysis, Methodology, Writing – review & editing, Validation. AL: Formal analysis, Methodology, Validation, Writing – review & editing. CB: Formal analysis, Methodology, Writing – review & editing. EW: Methodology, Writing – review & editing, Resources. NF: Conceptualization, Funding acquisition, Investigation, Methodology, Supervision, Writing – review & editing.
