# Corrigendum: Spatiotemporal analysis of 3D human iPSC-derived neural networks using a 3D multi-electrode array

**DOI:** 10.3389/fncel.2023.1340688

**Published:** 2023-12-07

**Authors:** Doris Lam, Heather A. Enright, Jose Cadena, Vivek Kurien George, David A. Soscia, Angela C. Tooker, Michael Triplett, Sandra K. G. Peters, Piyush Karande, Alexander Ladd, Chandrakumar Bogguri, Elizabeth K. Wheeler, Nicholas O. Fischer

**Affiliations:** ^1^Physical and Life Sciences Directorate, Lawrence Livermore National Laboratory, Livermore, CA, United States; ^2^Engineering Directorate, Lawrence Livermore National Laboratory, Livermore, CA, United States

**Keywords:** 3D multi-electrode array, microelectrode array, neural networks, electrophysiology, 3D culture, hiPSC, collagen

In the published article, there were errors in the legend for **Figure 5** as published. “ε” symbol was mistakenly used for “≥” and the color scheme for high and low synchrony was incorrect. The correct color scheme is purple for “high” synchrony and yellow for “low” synchrony. The corrected legend appears below.

“The effect of BIC, AP-5, and CNQX on synchronized neural network activity within a 3D neuron-astrocyte co-culture. **(A)** The heat map illustrates the average synchrony value per array within (bottom, middle 1, middle 2, and top) and between cross sections before and after the sequential addition of BIC, AP-5, and CNQX. **(B)** Overlay bar graph compares the average number of synchronized networks (or edges) detected within the 3D culture, independent of the electrode's position, across treatment conditions (e.g., baseline, BIC, AP-5+BIC, and CNQX+AP-5+BIC). Data was normalized to the total edges identified during BIC treatment (see Results for rationale). Edge activity has been categorized by the degree of synchrony: “high synchrony” has a 1-SPIKE distance ≥ 0.40 (purple), “low synchrony” has a 1-SPIKE distance < 0.40 (yellow), or inactive electrodes (white). Data is shown as mean ± SEM. **(C)** Scatter plot illustrates the shift in synchrony value for an active edge (dot) detected in BIC treatment (Reference, black line), its value before (e.g., baseline,), and after (e.g., AP-5 and CNQX) BIC treatment. The line of best fit was determined based on all available edges, categorized by the location within (i) or between (ii) cross sections, and the slope and intercept reported for each antagonist in brackets. **(D)** Scatter plot illustrates the shift in synchrony value for an active edge detected in BIC treatment (Reference, black line) based on its cross sectional position (e.g., bottom, middle 1, middle 2, and top graph) for baseline (i), AP-5 (ii), and CNQX (iii). The slope and intercept are reported based on the line of best fit for each cross section. Electrode data has been aggregated for 9 3D MEAs.”

In the published article, there was an error in Figure 2 as published. For panel B, the graph shown in burst per minute for the middle 2 cross section is incorrect. The corrected Figure 2 and its original caption appear below.

**Figure 2:**
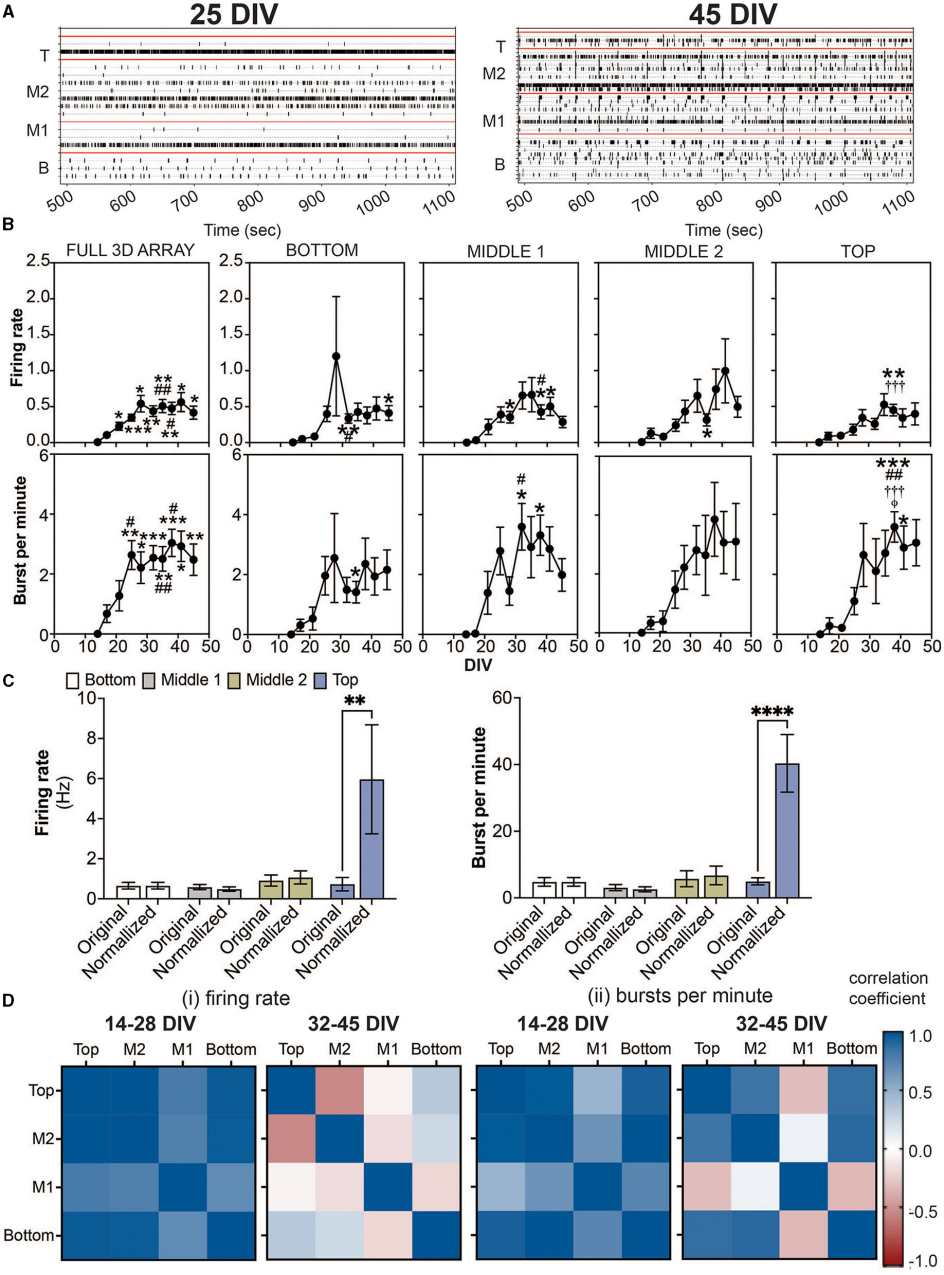
Detection of spikes and bursts within a 3D neuron-astrocyte co-culture monitored over 45 DIV. **(A)** Representative 10 min-raster plots of spiking and bursting activity within bottom, middle 1, middle 2 and top cross sections, delineated by the red line, of the flexible probes within the 3D MEA at 25 and 45 DIV. **(B)** Dot plots summarizes the firing rate (top row) and bursts per minute (bottom row) detected over the 30-min recordings. From left to right, graphs are shown with respect to the total activity across all electrodes within the (i.e.., full 3D array) and sections of the 3D array based on electrode positions (i.e., bottom, middle 1, middle 2 and top). Data is presented as the mean ± SEM for *n* = 15 wells and were analyzed using mixed model of two-way ANOVA with Tukey's *post hoc* test. Statistical significances (symbol) are observed for the feature of spike and burst activity when compared to 14 DIV (^*^), 17 DIV (#), 21 DIV (†), 25 DIV (‡), and 28 DIV (ϕ) at a level (number of symbols) of #*p* < 0.05, ## *p* < 0.01, ### *p* < 0.001. **(C)** Bar graph compares the original data for the firing rate (left) and bursts per minute (right) for each cross section, and when the average data is normalized by the cell density within each cross section, determined from (Figure 1C). Data is presented as the mean ± SEM for *n* = 15 wells and were analyzed using two-way ANOVA with Bonferroni's *post hoc* test. Statistical significance is reported at a level of ^**^p < 0.01, ^****^p < 0.0001 **(D)** Correlation coefficient matrices display the strong positive (correlation score closer to 1.0) and negative relationships (score closer to −1.0) between layers (top, middle 2, middle 1, and bottom) of the 3D culture for the mean firing rate (i) and burst per minute (ii) grouped by the growth phase (development) of the active culture (14–28 DIV), and plateau (maturation) phase (32–45 DIV).

In the published article, **Supplementary Table 1** was mistakenly not included in the publication.

The authors apologize for these errors and state that this does not change the scientific conclusions of the article in any way. The original article has been updated.

