# Supplementary material for: Spatiotemporal analysis of 3D human iPSC-derived neural networks using a 3D multi-electrode array
Source: Front Cell Neurosci. 2023 Nov 13;17:1287089. doi: 10.3389/fncel.2023.1287089 (PMC10679684; doi:10.3389/fncel.2023.1287089)
Supplement: Supplementary file 1 [file Data_Sheet_1.docx]

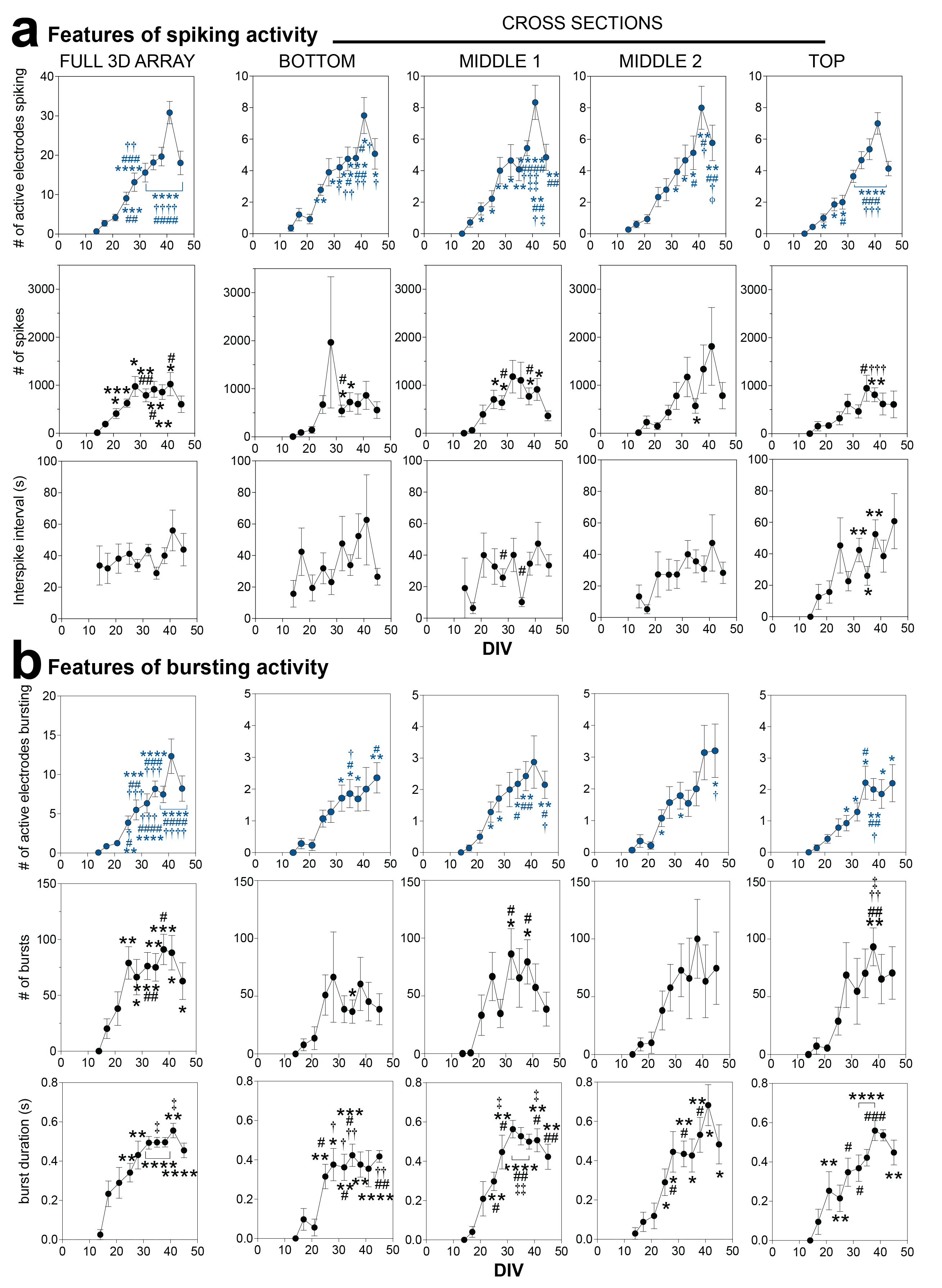


Supplementary Figure 1. **Additional features of spiking and bursting activity within a 3D neuron-astrocyte co-culture monitored over 45 DIV.** Plot summarizes the features of spiking (**a**) and bursting activity (**b**) from 3D MEA across 45 DIV, and includes: number of active electrodes for spiking, number of spikes, interspike interval, number of active electrodes bursting, number of bursts and burst duration. From left to right, graphs are shown with respect to the total activity across all electrodes within the array (e.g., full 3D array) and cross sections of the 3D array based on electrode positions (e.g., bottom, middle 1, middle 2 and top). Data is presented as the mean ± SEM for N=15 wells, and was analyzed using mixed model of one-way ANOVA with Tukey’s post hoc test. Statistical significances (symbol) are observed for the features of spike and burst activity (black) when compared to 14 DIV (*), 17 DIV (#), 21 DIV (†), 25 DIV (‡), and 28 DIV (φ) at a level (number of symbols) of #p <0.05, ## p<0.01, ### p<0.001, and #### p<0.0001.


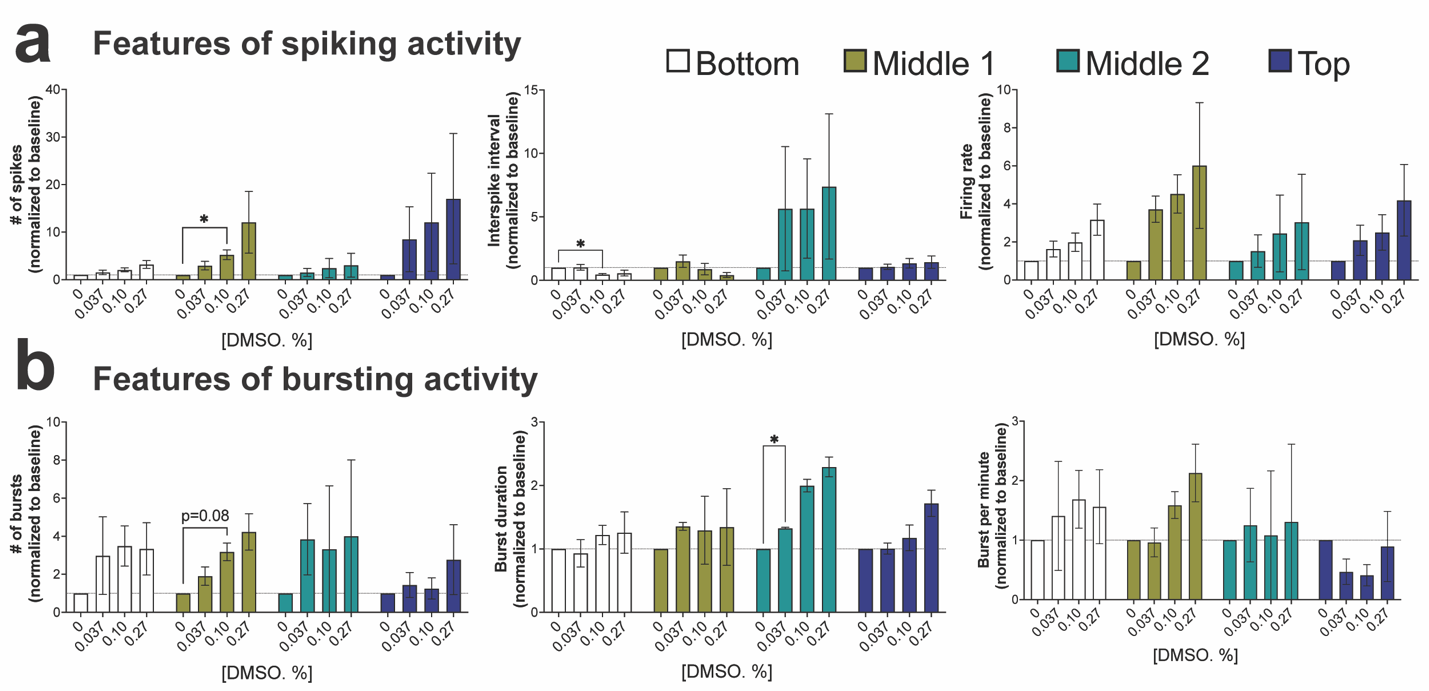


Supplementary Figure 2. **The effect of DMSO on features of spiking and bursting activity within a 3D neuron-astrocyte co-culture.** 3D co-cultures at 45 DIV were challenged with DMSO, vehicle control to the chemical challenged cultures. Features of spiking **(a)** and bursting **(b)** activity were normalized to baseline activity (before the addition of DMSO and is shown as a dotted line). Data is displayed as the mean ± SEM for N=3 arrays and was analyzed using two-way ANOVA with Dunnett’s post hoc test. Statistical significance was observed when comparing concentrations of DMSO to baseline (*) at a significance level of #p <0.05.
