# Supplementary material for: Spatiotemporal analysis of 3D human iPSC-derived neural networks using a 3D multi-electrode array
Source: Front Cell Neurosci. 2023 Nov 13;17:1287089. doi: 10.3389/fncel.2023.1287089 (PMC10679684; doi:10.3389/fncel.2023.1287089)
Supplement: Supplementary file 2 [file Table_1.docx]

**Supplementary table 1. p-value for the hypothesis test whose null hypothesis is that the slope of the treatment condition deviates from the slope of BIC, using Wald Test with t-distribution of the test statistic.**

|  | **p-value** | |
| --- | --- | --- |
| **Treatment condition** | **Networks within cross sections** | **Networks between cross sections** |
| **Baseline** | 3.21E-07 | 1.38E-01 |
| **AP-5 (+BIC)** | 2.98E-34 | 1.15E-12 |
| **CNQX (+AP-5 +BIC)** | 3.44E-17 | 2.22E-10 |
